# Pigmentation and Sporulation Are Alternative Cell Fates in *Bacillus pumilus* SF214

**DOI:** 10.1371/journal.pone.0062093

**Published:** 2013-04-25

**Authors:** Nicola Manzo, Blanda Di Luccia, Rachele Isticato, Enrica D’Apuzzo, Maurilio De Felice, Ezio Ricca

**Affiliations:** Department of Biology, Federico II University, Napoli, Italy; University of Padova Medical School, Italy

## Abstract

*Bacillus pumilus* SF214 is a spore forming bacterium, isolated from a marine sample, able to produce a matrix and a orange-red, water soluble pigment. Pigmentation is strictly regulated and high pigment production was observed during the late stationary growth phase in a minimal medium and at growth temperatures lower than the optimum. Only a subpopulation of stationary phase cells produced the pigment, indicating that the stationary culture contains a heterogeneous cell population and that pigment synthesis is a bimodal phenomenon. The fraction of cells producing the pigment varied in the different growth conditions and occured only in cells not devoted to sporulation. Only some of the pigmented cells were also able to produce a matrix. Pigment and matrix production in SF214 appear then as two developmental fates both alternative to sporulation. Since the pigment had an essential role in the cell resistance to oxidative stress conditions, we propose that within the heterogeneous population different survival strategies can be followed by the different cells.

## Introduction

Spore-forming *Bacilli* are Gram positive organisms characterized by the ability to differentiate the endospore (spore), a metabolically quiescent and extremely resistant cell type. The soil is generally indicated as the main habitat of *Bacilli*, however, spores have been found in many diverse environments, including rocks, dust, aquatic environments, and the gut of various insects and animals [1], [2]. Such a wide environmental distribution is facilitated by the spore ability to survive long-term absence of water and nutrients and withstand extreme habitats that would kill other cell types [3]. Survival is due to the peculiar structure of the spore that is formed by a dehydrated cytoplasm containing a condensed and inactive chromosome, and by a series of protective layers. An innermost layer is the peptidoglycan-rich cortex that is itself surrounded by additional layers of proteinaceous material, the coat and, in some species, the exosporium [4], [5]. Together these components protect the spore from UV radiation, extremes of heat or pH, exposure to solvents, hydrogen peroxide, toxic chemicals and lytic enzymes [3], [6]. In the presence of water and appropriate nutrients the spore starts germination, a fast process during which the protective structures are removed and resumption of vegetative cell growth is allowed [3], [4].

Spore formation is dependent upon environmental conditions that do not allow cell growth, such as a block of DNA replication and a decline of available nutrients [7]. In *Bacillus subtilis,* the model organism for spore formers, growing cells are mainly single and highly motile. When those dispersed cells reach the end of exponential growth they can follow alternative developmental pathways with some cells forming long chains, producing a polymeric matrix rich in sugars and proteins (matrix) and assembling into multicellular biofilms and others entering the irreversible program of spore formation [8], [9], [10]. Therefore, in dispersed cell populations matrix and spore production are mutually exclusive cell fates [8], [11] and are both bimodal processes in which cells follow either one or the other pathway [12], [13]. Both developmental cell fates are governed by a regulatory protein, Spo0A-P, that directly activates genes of the sporulation pathway [14] and indirectly acts on matrix synthesis, relieving the repression of genes for matrix production (*epsA-O* and *yqxM-sipW-tasA* operons) [15], [16], [17]. Two mechanisms cooperate to make sporulation and matrix production mutually exclusive: a metabolic control mediated by the intracellular levels of SpoOA-P and a chromosome copy number mechanism that prevents cells that have entered the sporulation pathway from expressing matrix genes [10]. Low levels of SpoOA-P induce matrix formation while high levels of the phosphoprotein block matrix formation and activate sporulation. Therefore, in a sporulation-inducing medium, in which SpoOA-P levels rapidly rise, cells enter sporulation instead of forming a biofilm. Conversely, in a medium in which SpoOA-P remains at low levels biofilm formation is promoted [10]. However, extracellular matrix production and sporulation are linked. KinD, a membrane histidine kinase which is part of the Spo0A phosphotransfer network, has been proposed to act as a checkpoint protein able to regulate the onset of sporulation by inhibiting Spo0A activity. KinD would alter its activity, depending on the presence or absence of the extracellular matrix, thus affecting the selective functionality on the master regulator Spo0A to regulate expression of genes involved in matrix production and sporulation [18]. Within a biofilm different cell types coexist and display a high degree of spatiotemporal organization with matrix-producing cells that ultimately differentiate into spores [8].

Another interesting feature of some *Bacilli* is the production of pigments. Isolates of several *Bacillus* species produce a wide variety of pigments, from spore-associated melanin-like molecules [19] to different types of carotenoids [20], [21]. In some cases, those carotenoids have been characterized and proposed to provide resistance to UV irradiation and reactive oxygen species [20], [21], [22], [23]. A pigmented strain of *Bacillus pumilus*, SF214, isolated from a marine sample, has been previously described [21]. SF214 is a moderate halophilic bacterium able to form a matrix and to produce an orange to red water-soluble pigment, i.e. a pigment that can not be partitioned into organic solvents but is retained in the aqueous phase [21]. The inability to partition this pigment into organic solvents, to resolve it by HPLC and to obtain characteristic carotenoid UV/VIS spectra, has precluded its definitive assignment as a carotenoid [21]. However, the spectral peak at 410 nm shown by aqueous extracts of SF214 [21] is likely to represent a protein-associated carotenoid, as previously described for carotenoproteins extracted from crawfishes [24].

Here we report that in SF214 pigment production is a highly regulated process that occurs during the stationary growth phase only in cells not devoted to spore formation. Thus SF214 pigment production appears as a bimodal phenomenon alternative to sporulation, parallel to matrix biosynthesis and essential to grant cell resistance to oxidative stress.

## Results

### Pigment Production is Dependent on Growth-phase, –temperature and –medium

**Figure 1:**
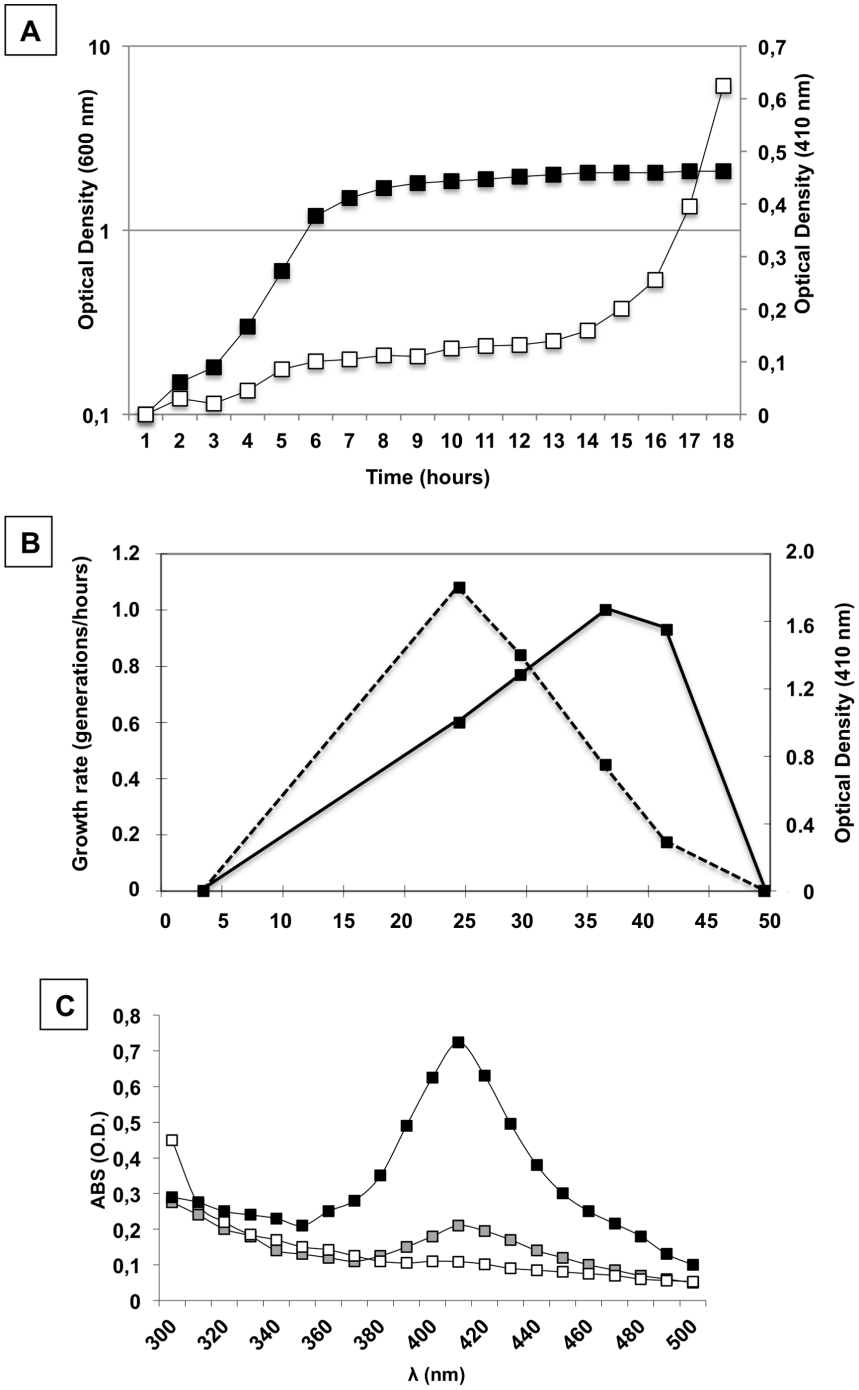
Characterization of growth and pigment production in *B.* *pumilus* SF214. (A) Growth curve of *B. pumilus* SF214 at 37°C in rich (LB) medium (black symbols) was determined by following over time the optical density of the culture at 600 nm. Pigment production (white symbols) was determined by measuring over time the optical density of 1 ml of cell-free culture supernatant at 410 nm. (B) Growth rate (continuous line) and pigment production (dashed line) of SF214 at different growth temperatures in rich (LB) medium. Pigment production was evaluated by measuring the optical density of 1 ml of cell-free culture supernatant at 410 nm after 24 hours of growth. (C) Absorbance spectrum between 300 and 500 nm of 360 µg of cell extracts of SF214 grown at 25°C in minimal (S7) (black symbol), rich (LB) (gray symbol) and sporulation-inducing (DS) medium.

Synthesis of the water-soluble pigment produced by SF214 is a strictly regulated process as it depends on the growth-phase, -temperature and -medium. Pigment production was shown to be strongly induced only 8–10 hours after that cells have entered the stationary growth phase at 37°C in rich (LB) medium (Fig. 1A). Although SF214 is a mesophilic bacterium and its optimal growth temperature is 37°C, the maximal production of the pigment was observed at 25°C (Fig. 1B). Compared with cells grown at 25°C a slightly decreased production of pigment was observed at 30°C, whereas more than 2-fold and about 6-fold decreased synthesis was observed at 37°C and at 42°C, respectively (Fig. 1B). The absorbance spectrum of cell extracts of SF214 between 300 and 500 nm [20] showed that cells grown at 25°C produced about 4-fold more pigment in a minimal (S7; black symbols in Fig. 1C) than in a rich (LB; gray symbols in Fig. 1C) medium, while in a sporulation-inducing (DS; white symbols in Fig. 1A) medium the synthesis of pigment was almost abolished.

### Heterogeneity of Pigment Production

**Figure 2:**
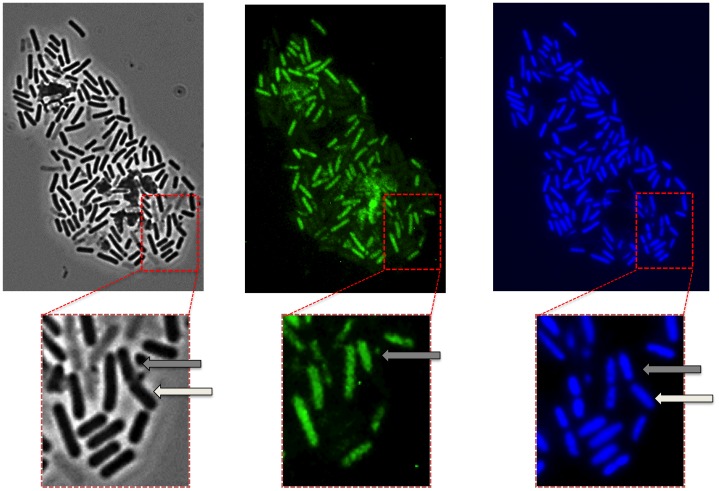
Phase contrast and fluorescence microscopy analysis of SF214. Observation of the same microscopy field by phase contrast (left), autofluorescence (middle) and DAPI staining (right). The same section of each panel is enlarged. The arrows in the enlarged sections point to a doublet of cells, still partially attached and deriving from the same mother cell, in which only one cell (grey arrows) is autofluorescent.

Previous reports have shown that carotenoids produced by the yeast *Phaffia rhodozyma* [25] or the halotolerant green alga *Dunaliella salina* [26] autofluoresce and that such property can be used to follow carotenoid production by fluorescence microscopy. We found that the water-soluble pigment of SF214 is also autofluorescent and that the fluorescence is not localized but rather diffuse in the cell cytoplasm. Interestingly, in the cell culture only some of the cells are fluorescent. Fig. 2 shows a representative microscopy field observed by phase contrast (left) and fluorescent microscopy either following the autofluorescence (middle) or after DAPI-staining (right). The enlarged panels of Fig. 2 clearly show that only some of the DAPI-stained cells were autofluorescent. The number of autofluorescent cells varied with the growth conditions (see below) but ranged between 20% in exponentially growing cells to 80% in stationary cells. Ghost-like cells, negative to DAPI staining and showing some autofluorescence were not considered. It is interesting to observe in Fig. 2 a doublet of cells (white and grey arrows in the enlarged sections). Those two cells seem to be still partially attached and to derive from the same mother cell, following the last round of division before stationary phase. Only one of them (grey arrows) has switched to the “pigment state” and is autofluorescent.

**Figure 3:**
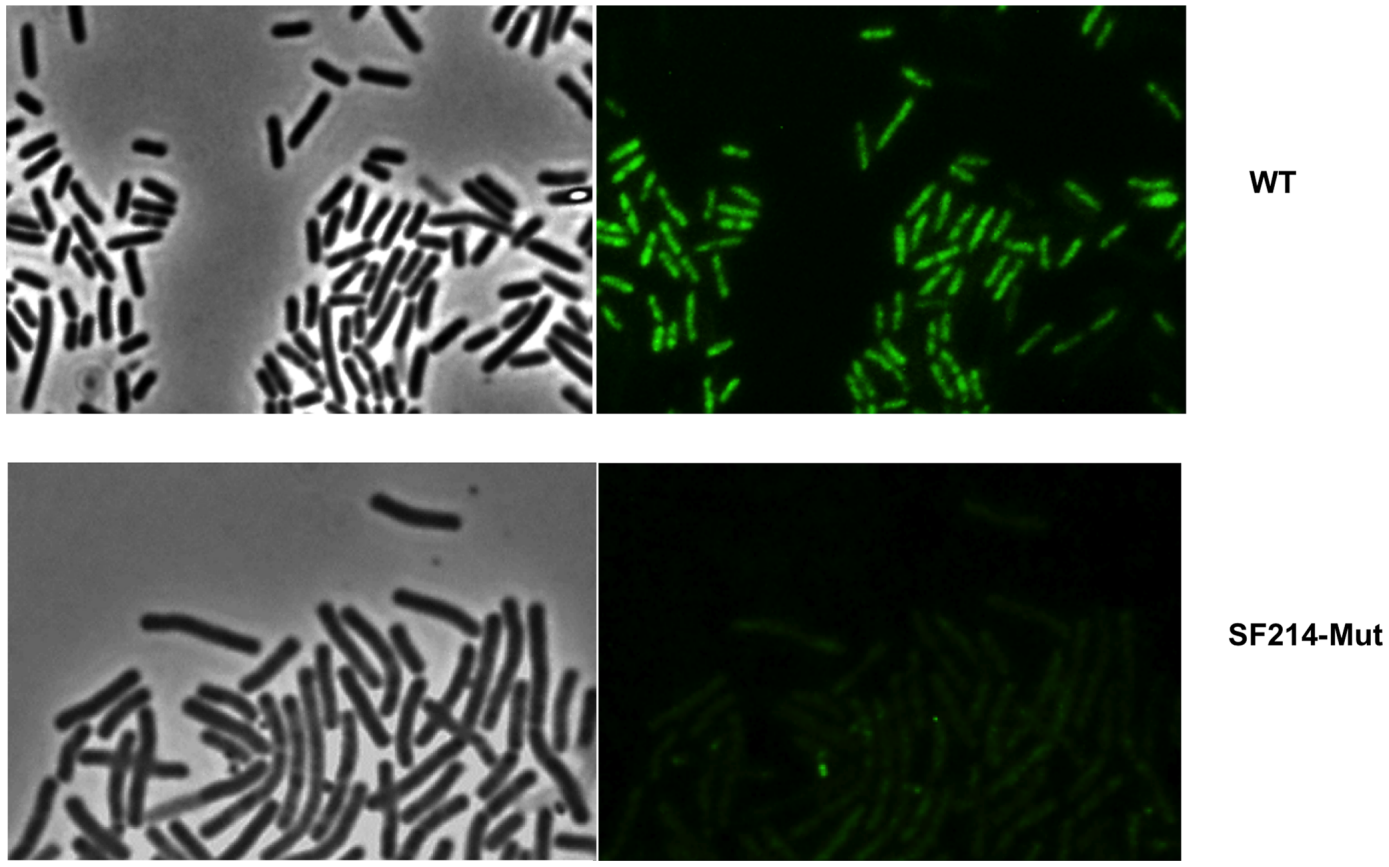
Microscopy analysis of SF214 and of its unpigmented mutant. Microscopy analysis of SF214 and of its unpigmented mutant (SF214-Mut). For each strain the same microscopy field is shown by phase contrast (left) and autofluorescence (right). The same conditions of exposure were used for the two microscopy fields.

**Figure 4:**
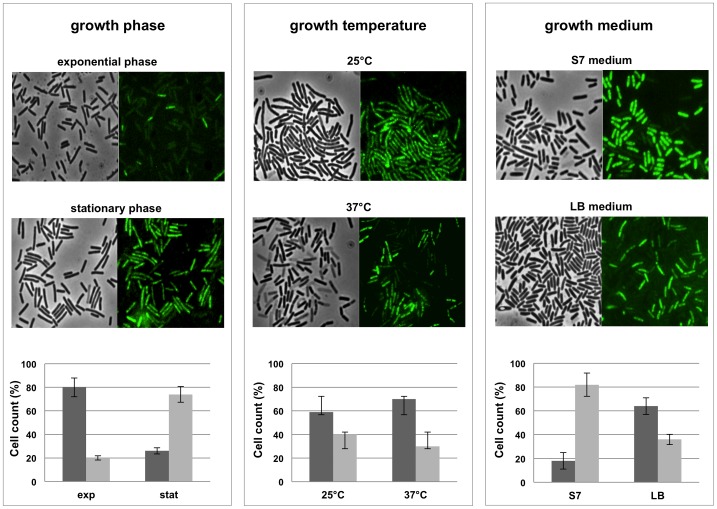
Autofluorescence at different growth conditins. Microscopy fields of SF214 cells grown at different conditions and observed by phase contrast and autofluorescence and compared to assess the proportion of fluorescent vs not fluorescent cells. Left panel: exponential vs. stationary growth phase (in LB medium at 37°C); middle panel: 25°C vs. 37°C as growth temperature (in LB medium for 24 hours); right panel: minimal (S7) vs. rich (LB) growth medium (stationary cells grown at 25°C). For each panel a graph reports the percentage of fluorescent (gray bars) vs. not fluorescent (dark gray bars) cells. For each condition a total of 1.000 cells from five different microscopy fields were counted. Spores and cells containing a prespore were not counted.

Two lines of evidence support our conclusion that the observed autofluorescence was actually due to the water-soluble pigment: i) unpigmented *Bacilli* (including other isolates of *B. pumilus*) (not shown) and an unpigmented mutant of SF214 (described below) did not show any fluorescence under identical experimental conditions (Fig. 3); ii) the number of autofluorescent cells varied consistently with the variations of pigment production observed at various growth-phase, -temperature and -medium. As shown in Fig. 4, the number of fluorescent cells was higher in a stationary than in an exponential cultures (left panels), in cells grown at 25°C than in cells grown at 37°C (middle panels) and in cells grown in minimal (S7) than in rich medium (LB) (right panels). For each condition considered in Fig. 4, different microscopy fields were analyzed and over 1,000 cells for each condition counted. This analysis indicated that the increased production of pigment observed depending upon growth-phase, -temperature and -medium is not due to a higher production of carotenoid by each producing cell but rather to an increased proportion of cells able to produce the pigment. Restriction of pigment synthesis to a subpopulation of cells indicates that late stationary cultures of SF214 contain a heterogeneous population of cells and that pigment formation is a bimodal process.

### Pigment Synthesis Only Occurs in Cells not Devoted to Sporulation

**Figure 5:**
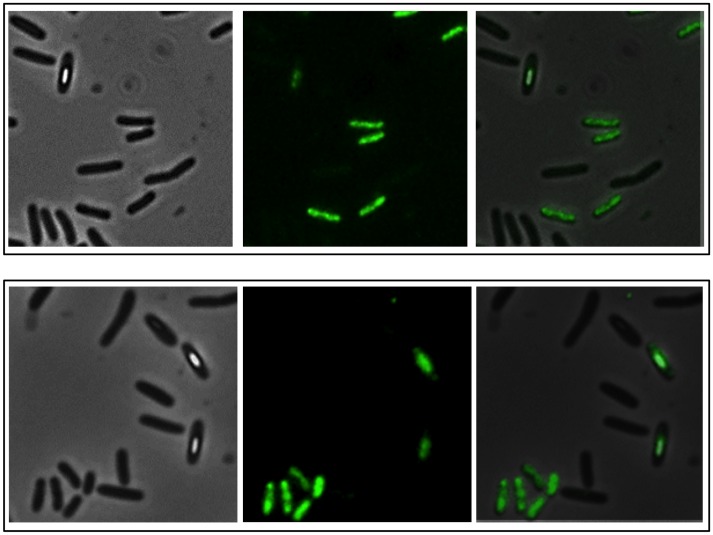
Autofluorescence of sporulating cells. The same microscopy field observed by phase contrast (left), autofluorescence (middle). The right panel reports the merge of phase contrast and autofluorescence images. Cells were grown in rich (LB) medium for 15 hours.

Free spores as well as immature spores still contained within the mother cells are known to autofluoresce [27]. We observed that the fluorescence of sporangia containing an almost mature spore was always limited to the prespore. Fig. 5 shows a representative microscopy field with sporulating cells of SF214 observed by phase contrast (left), autofluorescence (middle) and the merge (right): while only some cells autofluoresced with a fluorescence diffused in the cytoplasm, fluorescence associated to sporangia containing an almost mature spore was confined to the forming spore, as no fluorescence was visible within the cytoplasm. This observation, together with experiments reported in Fig. 1C indicating that when grown in a sporulation-inducing (DS) medium SF214 cells did not produce the pigment, suggests that pigment production in *B. pumilus* SF214 is mutually exclusive with spore formation.

**Figure 6:**
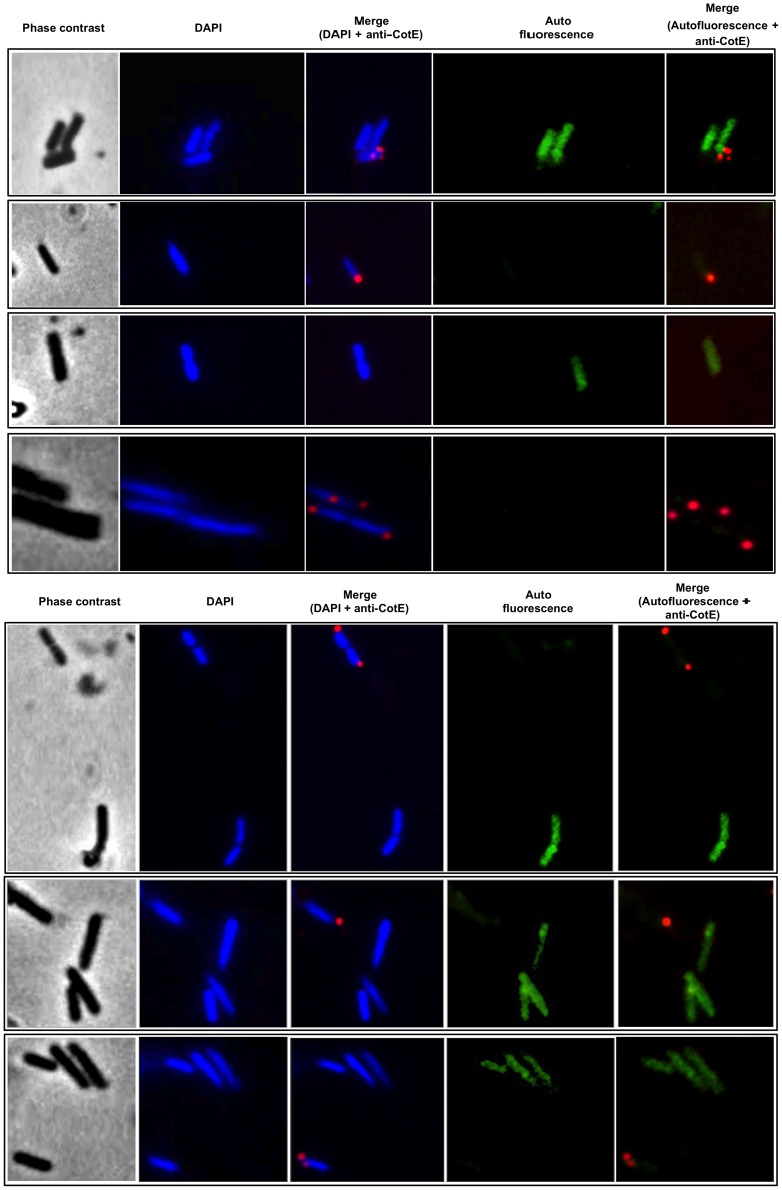
Fluorescence and immunofluorescence microscopy with anti-CotE antibody. Microscopy analysis of cells from different fields observed by phase contrast, DAPI-staining, immunofluorescence with anti-CotE primary antibody and Texas Red conjugated secondary antibody. Merged panels of DAPI-immunofluorescence and autofluorescence-immunofluorescence are shown.

To better address this point we analyzed SF214 cells by pigment-driven autofluorescence (green) and by immunofluorescence due to anti-CotE primary antibody and fluorescent secondary antibody (red). CotE is a spore coat protein [28], produced early during sporulation, known to localize on the spore surface [27]. For our analysis antibody raised against CotE of *B. subtilis* were used [29]. In a preliminary experiment this antibody was shown to specifically react against a protein of *B. pumilus* SF214 corresponding in size to CotE of *B. subtilis* (Fig. S1). Fig. 6 reports representative microscopy fields of fluorescence and immunofluorescence microscopy of SF214 cells grown in LB at 37°C up to the early stationary growth phase. In this analysis we observed that, similarly to what observed in *B. subtilis* [27], *B. pumilus* CotE is localized around the forming spore, and that cells recognized by the anti-CotE antibody were all not autofluorescent. We never observed yellow cells, which would have been indicative of cells producing the pigment (green signal) and the spore-specific protein CotE (red signal) (see the merged panels of Fig. 6 for some examples). Therefore, based on the experiments of Figs. 5 and 6 we conclude that pigment synthesis and sporulation are alternative developmental pathways and occur in different cell subpopulations.

### Matrix Synthesis Occurs Only in a Subpopulation of Pigmented Cells

**Figure 7:**
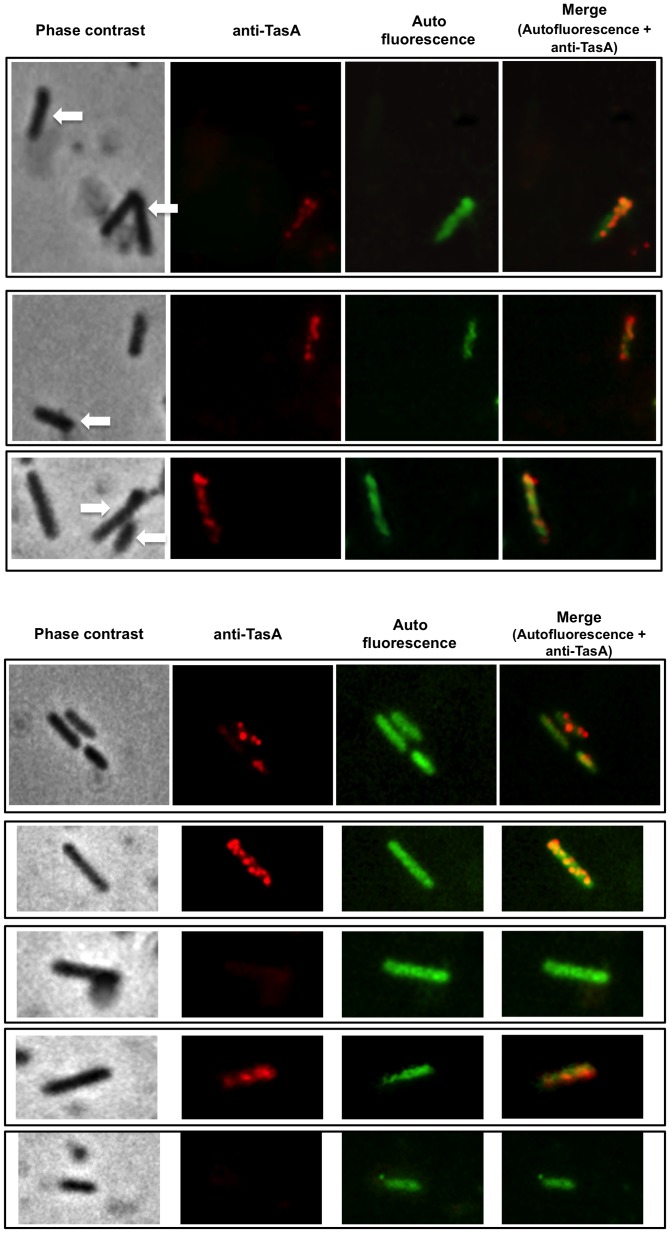
Fluorescence and immunofluorescence microscopy with anti-TasA antibody. Microscopy analysis of cells from different fields observed by phase contrast, immunofluorescence with anti-TasA primary antibody and Texas Red conjugated secondary antibody and autofluorescence. Merged panels of autofluorescence-immunofluorescence are shown. Panel A reports examples of cells that are not autofluorescent and that are also not recognized by anti-TasA, indicated by arrows. Panel B focuses on examples of autofluorescent cells, with some recognized (yellow in the merge) and some not recognized by anti-TasA antibody.

In *B. subtilis* sporulation and matrix formation are alternative developmental programmes [11]. Since SF214 also forms a matrix [21] we verified whether also in this bacterium matrix formation and sporulation are alternative and whether matrix and pigment synthesis can occur in the same cells. To this aim we analyzed SF214 cells by pigment-driven autofluorescence (green) and by immunofluorescence due to anti-TasA primary antibody and fluorescent secondary antibody (red). TasA is a major protein component of the *B. subtilis* biofilm [15], encoded by the third gene of the *yqxM-sipW-tasA* operon [16], [30]. For our analysis we used antibody raised against TasA of *B. subtilis* (a gift of A. Driks). Preliminary experiments showed that a protein homologous to TasA of *B. subtilis* can be extracted from spores of strain SF214 and that this protein is recognized by the anti-TasA antibody (Fig. S2). The homology with the protein of SF214 starts at position 24 of TasA, which corresponds to the first amino acid residue of the mature form of TasA after the proteolytic maturation of pre-TasA [30], [31] (Fig. S2). Fig. 7 reports representative fields of fluorescence and immunofluorescence microscopy of SF214 cells grown in minimal (S7) medium at 25°C up to the early stationary phase. This analysis showed that: i) cells that were not autofluorescent and therefore devoted to sporulation (indicated by white arrows in Fig. 7A) were never recognized by the anti-TasA antibody, and ii) only about 80% of the autofluorescent cells (from a total of approx. 1500 cells counted in 6 different microscopy fields) were recognized by anti-TasA antibody (yellow cells in Fig. 7). Panel B of Fig. 7 shows some examples of autofluorescent cells that are not recognized by anti-TasA antibody. These results indicate that as in *B. subtilis* also in *B. pumilus* SF214 sporulation and matrix formation are alternative and that matrix synthesis occurs only in a subpopulation of pigmented cells.

### The Pigment of SF214 is Essential for Cell Resistance to Hydrogen Peroxide

In non-photosynthetic organisms pigments have been associated to cell resistance to UV irradiation and reactive oxygen species [21], [22], [23]. To analyze the role of the SF214 pigment we isolated an unpigmented mutant after nitrosoguanidine (NTG) mutagenesis [32] (Fig. S3). To this aim mid-exponential phase cells were incubated for different times with 10 µg of NTG and the percentage of survival assessed by CFU determination (Fig. S3B). To minimize the possibility to have mutants carrying multiple mutations, we only analyzed cells exposed to NTG for the shortest time. NTG-treated cells were then diluted, plated and checked for pigmentation after 36 hours of incubation at 25°C. One unpigmented mutant, SF214-Mut, was chosen for further analysis. Although we could not isolate the mutation responsible for loss of pigmentation, as several attempts to transform SF214 with either plasmid or chromosomal DNA resulted unsuccessful (not shown), we were able to show that the unpigmented phenotype reverted spontaneously at a frequency of 1 clone out of 10^9^, thus suggesting that the NTG treatment had not produced multiple mutations. Analysis of the aqueous extracts showed that the mutant does not produce any molecule able to adsorb at 410 nm (Fig. S4) and, consistently, a fluorescence microscopy analysis showed that no fluorescent cells were present in a stationary phase culture of the unpigmented mutant (Fig. 3).

**Figure 8:**
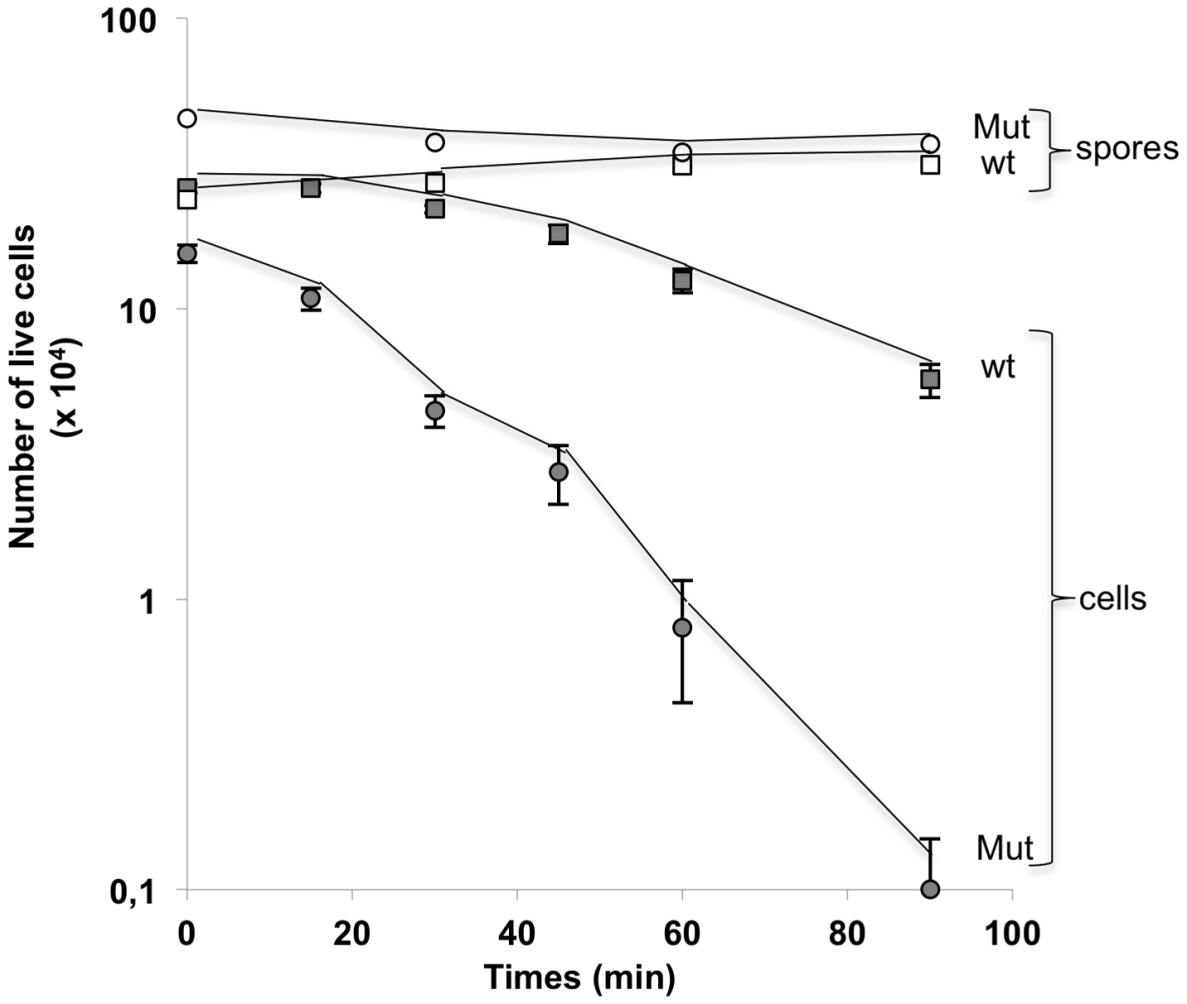
Cell and spore survival after treatment with H_2_O_2_. Cells and spores were treated with 30 mM H_2_O_2_ for various times. For each time point the CFU of cells (gray symbols) and spores (white symbols) of the wild type (squares) and the mutant (circles) strains was obtained by plating on LB plates and incubation for 24 hours at 37°C.

SF214 and its unpigmented derivative were used to analyze the cell response to hydrogen peroxide. Cells of the two strains were grown at 25°C in minimal (S7) liquid medium and collected 10 hours after the entry into stationary phase. Cells were then incubated with 30 mM hydrogen peroxide and analyzed for viability after various incubation times. While wild type cells were all viable after exposure to hydrogen peroxide for up 30 minutes and showed a reduced viability only after 45, 60 and 90 minutes of treatment, the unpigmented mutant showed a clear decrease of viability at all incubation times (Fig. 8). In a parallel experiment spores of both strains were totally resistant to the hydrogen peroxide treatment at all time points tested (Fig. 8). Results of Fig. 8 confirm that the pigment has a role in the response of vegetative cells to oxidative stress. Spores do not contain the pigment but are totally resistant to hydrogen peroxide due to other, pigment-independent mechanisms [6], [33].

## Discussion

The main result of this report is the observation that pigment production in SF214, a marine isolate of *B. pumilus,* is a bimodal phenomenon alternative to sporulation. SF214 cells in stationary growth phase form a heterogeneous population able to follow diverse developmental fates. Some cells start the sporulation programme while others produce the pigment. Only a subpopulation of pigmented cells also produces a matrix. This is reminiscent of the situation found in *B. subtilis*. Seminal studies performed using *B. subtilis*, the model organism for spore formers, have shown that in dispersed cell populations spore formation, matrix production, competence to acquire external DNA and production of extra-cellular proteases are all bimodal processes [9], [10], [12]. Spore and matrix formation appear as alternative developmental pathways with some cells producing a matrix and others entering the irreversible program of spore formation [8], [9], [10]. In addition to those two also other cell fates are alternative in *B. subtilis:* within a biofilm, only a subpopulation of *B. subtilis* cells produce surfactin but, while surfactin-producers do not respond to their own surfactin, other cells do and become matrix producers. In this case, individual *B. subtilis* cells simultaneously expressing genes for both surfactin and matrix synthesis have never been observed [11]. These two subpopulations do not include the entire population and the rest of the cells that do not differentiate as surfactin or matrix producers probably originate the other cell types known to be present in *B. subtilis* populations [13]. In this frame each differentiation fate sets the stage for a subsequent cell type. For example, within biofilms matrix-producing cells are initially predominant and later differentiate and become spores [8]. By analogy, we propose that *B. pumilus* SF214 dispersed stationary cells also form a heterogeneous population able to follow diverse developmental fates. Some cells enter the irreversible sporulation cycle forming the highly resistant but metabolically quiescent spore while other cells follow a different survival strategy and produce a pigment able to protect the cell from oxidative conditions. Cell diversification and the ability to develop different survival strategies in *B. pumilus* SF214 can then be viewed as a risk spreading (or bet hedging) strategy. Such stochastic switches between phenotypic states have been found in diverse organisms ranging from bacteria to humans and are considered among the earliest evolutionary solutions to adapt and facilitate persistence in fluctuating environments [34].

Only a subpopulation of pigment-producing cells forms an extracellular matrix. It is not clear whether matrix-production can also be viewed as a survival strategy in specific environments. However, the existence of more than two developmental cell fates is not surprising but rather expected on the base of the multiple cell types previously observed in *B. subtilis* [7], [13].

An additional result of this work is the observation that pigment formation is a highly regulated process. Growth conditions affect pigment synthesis most probably regulating the number of cells that become able to synthesize the pigment. This conclusion is supported by the number of fluorescent vs. not fluorescent cells in diverse microscopy fields (Fig. 4). Although our analysis does not allow us to assess the amount of pigment synthesized at a single-cell level in the various conditions, it clearly shows that a regulation is exerted when the single stationary cell turns its fate towards either sporulation or pigment synthesis.

Strain SF214 of *B. pumilus* is a field isolate and our attempts to genetically manipulate it have been so far unsuccessful. Several attempts to transform SF214 with chromosomal DNA of an antibiotic-resistant strain of *B. pumilus* or with a non replicative plasmid have all been unsuccessful. SF214 contains a large natural plasmid. We obtained a cured strain which did not show apparent phenotypic differences from SF214 but that was still refractory to transformation. The impossibility to manipulate SF214 has so far impaired a deeper molecular analysis of the various developmental fates of SF214 and of the regulatory proteins involved. A future challenging task will be to verify whether the master regulator Spo0A, known to control matrix formation and sporulation, as well as other cell fate regulators of *B. subtilis* such as ComX and SinI/R, is also involved in pigment development in *B. pumilus.*

## Methods

### Bacterial Growth Conditions and Spore Induction and Purification

Bacilli were grown either in LB medium (for 1 l: 10 g Bacto-Tryptone, 5 g Bacto-yeast extract, 10 g NaCl, pH 7.0) or in Difco-Sporulation-inducing (DS) medium or in minimal S7 medium (50 mM MOPS, 10 mM (NH_4_)2SO_4_, 5 mM potassium phosphate pH 7.0, 2 mM MgCl_2_, 0.9 mM CaCl_2_, 50 µM MnCl_2_, 10 µM ZnCl_2_, 5 µM FeCl_3_, 2 µM thiamine hydrochloride, 20 mM sodium glutamate, 1% glucose, 0.1 mg/ml phenylalanine, 0.1 mg/ml tryptophan) in aerobic conditions. For spore production cells were grown in DS medium in aerobic conditions for 48 hours [32]. Spores were collected by centrifugation and purified by repeated washes and lysozyme treatment, as previously reported [35]. The *B. subtilis* strains used as reference were PY79 (wild type) [36] and the isogenic BZ213 (*cotE::cat*) [28].

### Pigment Extraction and Detection

For pigment extraction, cultures were centrifugated at 7000 rpm for 10 minutes. The cell pellet was suspended in a lysis buffer (50 mM Tris-HCl pH 7.5, 1 mM DTT, 0.1 mM PMSF, 10% glycerol) and sonicated at 4°C for 10 min (30 sec. ON and 30 sec. OFF). The pellet was completely removed by centrifugation at 13000 rpm for 15 minutes. Protein concentration of the various extracts was determined spectrophotometrically and aliquots of identical protein concentration used to determine the adsorbance spectrum between 300 and 550 nm, as previously reported [21].

### Hydrogen Peroxide Assays

Vegetative cells and spores were diluted to a concentration of approximately 10^8^ CFU/ml in PBS, and 1 ml of the cell suspensions placed in a 1.5 ml microcentrifuge tube. 30 mM H_2_O_2_ (Sigma) was added to the cell suspensions at the concentration of 30 mM. Spores or cell suspensions were incubated at room temperature with continous gentle mixing. After various incubation times 100-µl samples were removed, immediately diluted, plated onto LB agar plate and incubated in order to determine the number of colonies.

### Fluorescence and Immunofluorescence Microscopy

For autofluorescence and DAPI staining 200 µl aliquots of cell culture were centrifuged (2 min 6,000 g) and cells resuspended in 20 µl of phosphate-buffered saline (PBS, pH 7.4). Only for the DAPI staining PBS contained 0.1 µg/ml of 4′,6-diamidino-2-phenylindole dihydrochloride (DAPI). Six microliters of each sample were placed on microscope slides and covered with a coverslip previously treated for 30 seconds with poly-L-lysine (Sigma). Samples were observed with an Olympus BX51 fluorescence microscope using a Fluorescein-Isothiocyanate (FITC) or DAPI filters to visualize the fluorescence of the cells. Typical acquisition times were 2000 ms for autofluorescence and 100 ms for DAPI and the Images were captured using a Olympus DP70 digital camera and processed.

Immunofluorescence was performed essentially as described by Azam et al (2000) [37], with a few modifications. Bacteria were fixed for 1 hour at room temperature in 80% methanol, washed, briefly treated with lysozyme and fixed to poly-L-lysine-treated coverslip slides to improve micrographs resolution. The coverslips were air dried and pretreated with 5% (w/v) dried milk in PBS, prior to incubation overnight a 4°C with the primary antibodies. In particular, a 1∶400 diluition of anti-CotE (raised in mouse) and a 1∶300 dilution for anti-TasA (raised in rabbit) were used. After ten washes, the samples were incubated with a 1000-fold diluted specific secondary antibody conjugates with Tetramethyl Rhodamine, TRITC (Santa Cruz Biotechnology, Inc.) for 2 hours at room temperature in the dark. After ten washes the coverslips were covered with one drop (30 µl) of Component C (Slow Fade: Molecular Probe S-2828) containing 0.1 µg/mL of DAPI. After 5 minutes the liquid was aspirated and the coverslips mounted onto microscope slides adding one drop of Component A (Slow Fade: Molecular Probe S-2828). The microscope slides were analyzed as described above.

## Supporting Information

Figure S1 **Western blot analysis of spore coat proteins of SF214 with anti-CotE antobody.** Purified spores were extracted by SDS-DTT treatment as previously reported (Nicholson and Setlow, 1990), fractionated on 12% SDS-PAGE and blotted on a PVDF membrane. The membrane was reacted with antibody raised against the CotE protein of *B. subtilis* (Isticato et al., 2010), then reacted against HRP-conjugated secondary antibody and visualized by the ECL method. Coat proteins of a wild type and an isogenic mutant lacking CotE of *B. subtilis* were used as positive and negative control, respectively.
(TIF)

Figure S2 **Characterization of TasA of** ***B. pumilus*** **SF214.** (A) An abundant protein extracted from SF214 spores and corresponding in size to TasA of *B. subtilis* (28 kDa) was transferred to a PVDF membrane and subjected to the Edman degradation reaction. The determined N-terminal 20 residues are reported. A Blast analysis identified the SF214 protein as an homolog of TasA of *B. subtilis* with the homology starting at position 24 of the *B. subtilis* protein. (B) Western blot analysis of spore coat proteins of SF214. Purified spores were extracted with SDS-DTT as previously reported (Nicholson and Setlow, 1990), fractionated on 12% SDS-PAGE and blotted on a PVDF membrane. The membrane was reacted with antibody raised against the TasA protein of *B. subtilis*, then reacted against HRP-conjugated secondary antibody and visualized by the ECL method. Coat proteins of a wild type strain (PY79) of *B. subtilis* were also used.
(TIF)

Figure S3 **Isolation of an unpigmented mutant of SF214.** (A) SF214 wild type and unpigmented mutant (Mut) on a LB plate grown at 25°C for 48 hours. (B) Survival of SF214 after treatment with 10 mg of NTG for various times. Mid-exponential cells were treated with NTG, washed twice, diluted, plated on LB plates and incubated at 37°C for 36 hours.
(TIF)

Figure S4 **Pigment production in SF214 and its unpigmented mutant.** Adsorbance spectrum between 300 and 500 nm of 360 mg of cell extracts of SF214 wild type (black symbols) and unpigmented mutant SF214-Mut (white symbols). Cells of both strains were grown at 25°C for 24 hours.
(TIF)
