# Supplementary figures and images for: Pigmentation and Sporulation Are Alternative Cell Fates in Bacillus pumilus SF214
Source: PLoS One. 2013 Apr 25;8(4):e62093. doi: 10.1371/journal.pone.0062093 (PMC3636246; doi:10.1371/journal.pone.0062093)

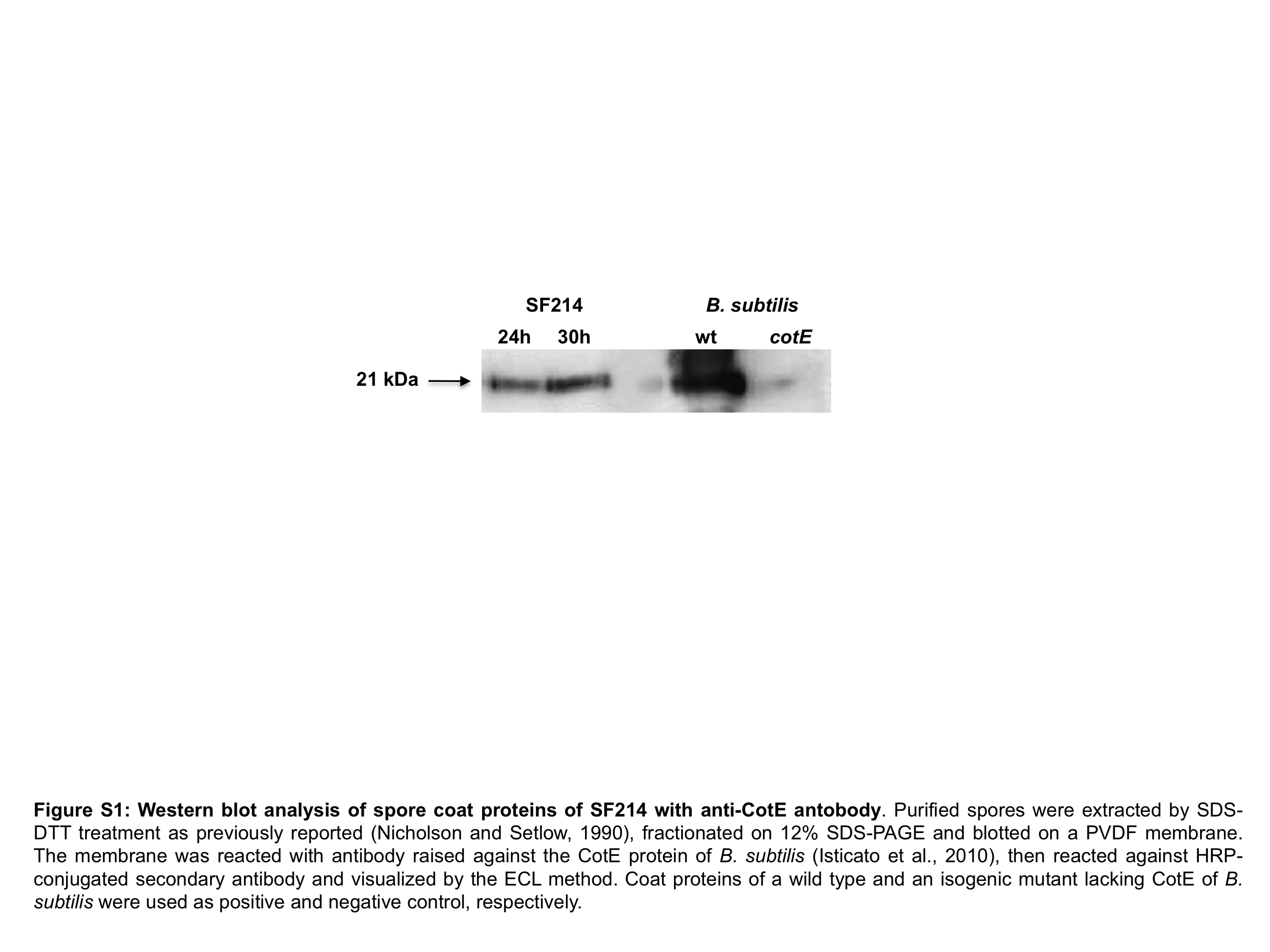

Supplement: Figure S1 — Western blot analysis of spore coat proteins of SF214 with anti-CotE antobody. Purified spores were extracted by SDS-DTT treatment as previously reported (Nicholson and Setlow, 1990), fractionated on 12% SDS-PAGE and blotted on a PVDF membrane. The membrane was reacted with antibody raised against the CotE protein of B. subtilis (Isticato et al., 2010), then reacted against HRP-conjugated secondary antibody and visualized by the ECL method. Coat proteins of a wild type and an isogenic mutant lacking CotE of B. subtilis were used as positive and negative control, respectively. (TIF) [file pone.0062093.s001.tif]

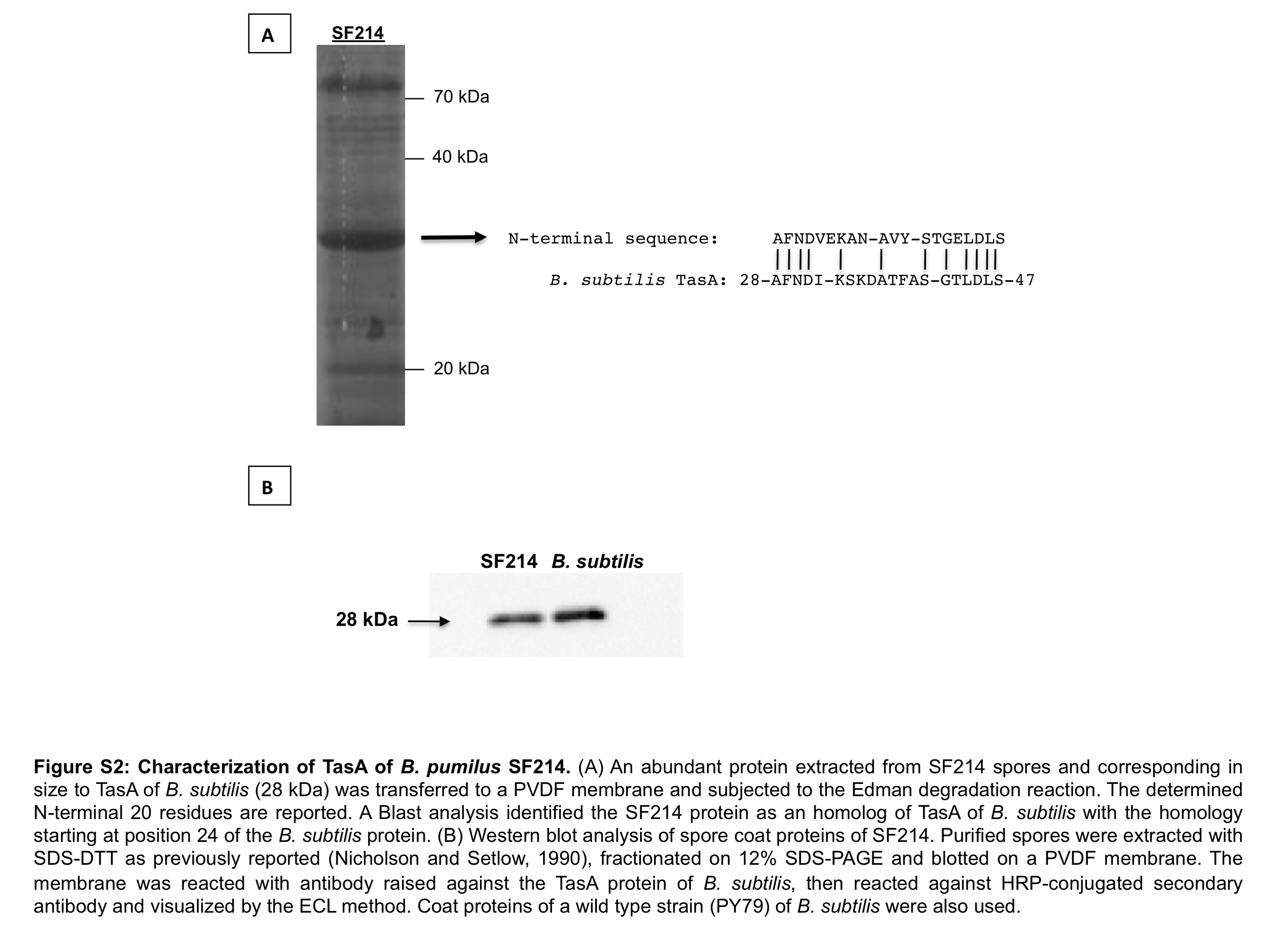

Supplement: Figure S2 — Characterization of TasA of B. pumilus SF214. (A) An abundant protein extracted from SF214 spores and corresponding in size to TasA of B. subtilis (28 kDa) was transferred to a PVDF membrane and subjected to the Edman degradation reaction. The determined N-terminal 20 residues are reported. A Blast analysis identified the SF214 protein as an homolog of TasA of B. subtilis with the homology starting at position 24 of the B. subtilis protein. (B) Western blot analysis of spore coat proteins of SF214. Purified spores were extracted with SDS-DTT as previously reported (Nicholson and Setlow, 1990), fractionated on 12% SDS-PAGE and blotted on a PVDF membrane. The membrane was reacted with antibody raised against the TasA protein of B. subtilis, then reacted against HRP-conjugated secondary antibody and visualized by the ECL method. Coat proteins of a wild type strain (PY79) of B. subtilis were also used. (TIF) [file pone.0062093.s002.tif]

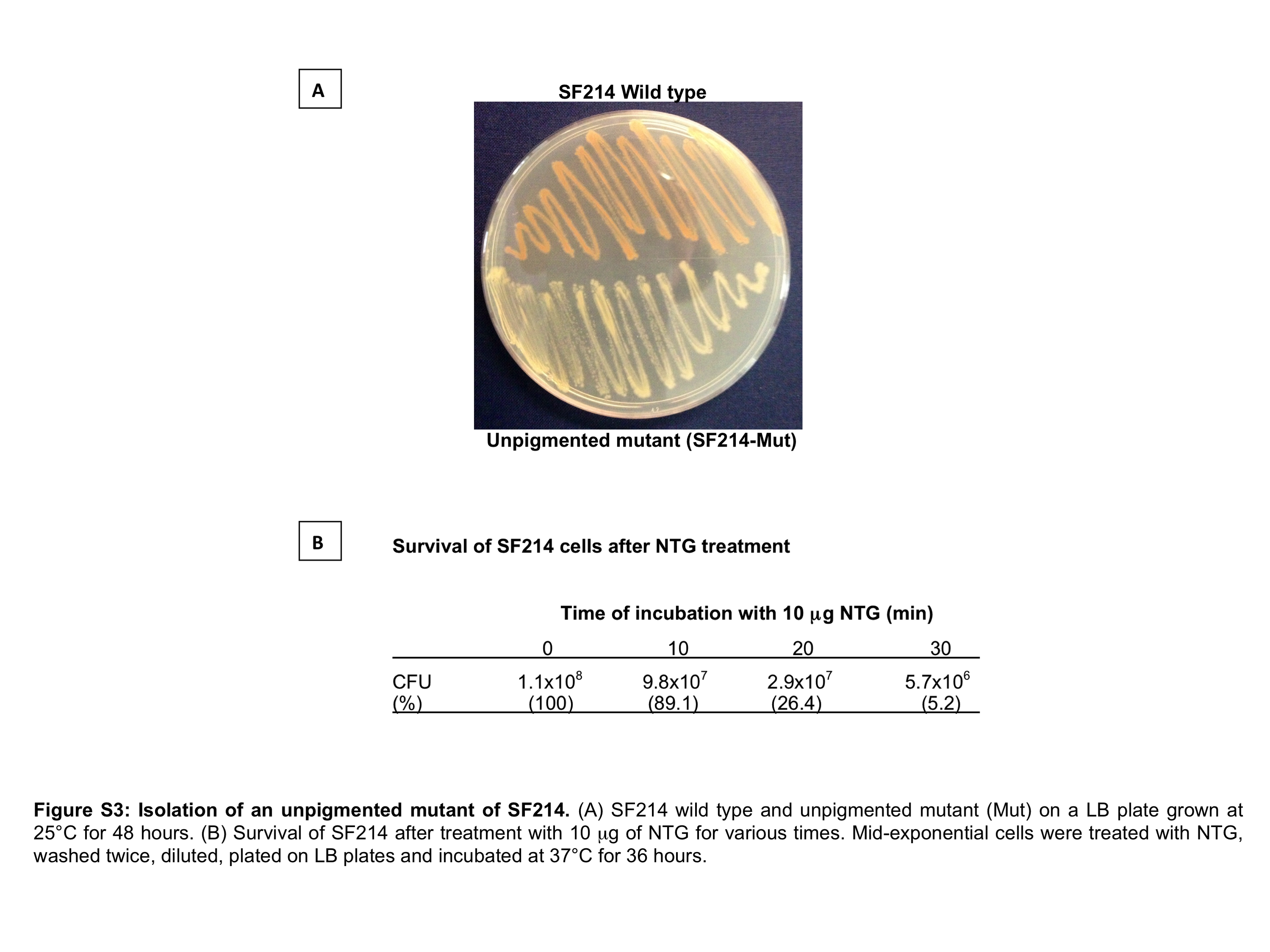

Supplement: Figure S3 — Isolation of an unpigmented mutant of SF214. (A) SF214 wild type and unpigmented mutant (Mut) on a LB plate grown at 25°C for 48 hours. (B) Survival of SF214 after treatment with 10 mg of NTG for various times. Mid-exponential cells were treated with NTG, washed twice, diluted, plated on LB plates and incubated at 37°C for 36 hours. (TIF) [file pone.0062093.s003.tif]

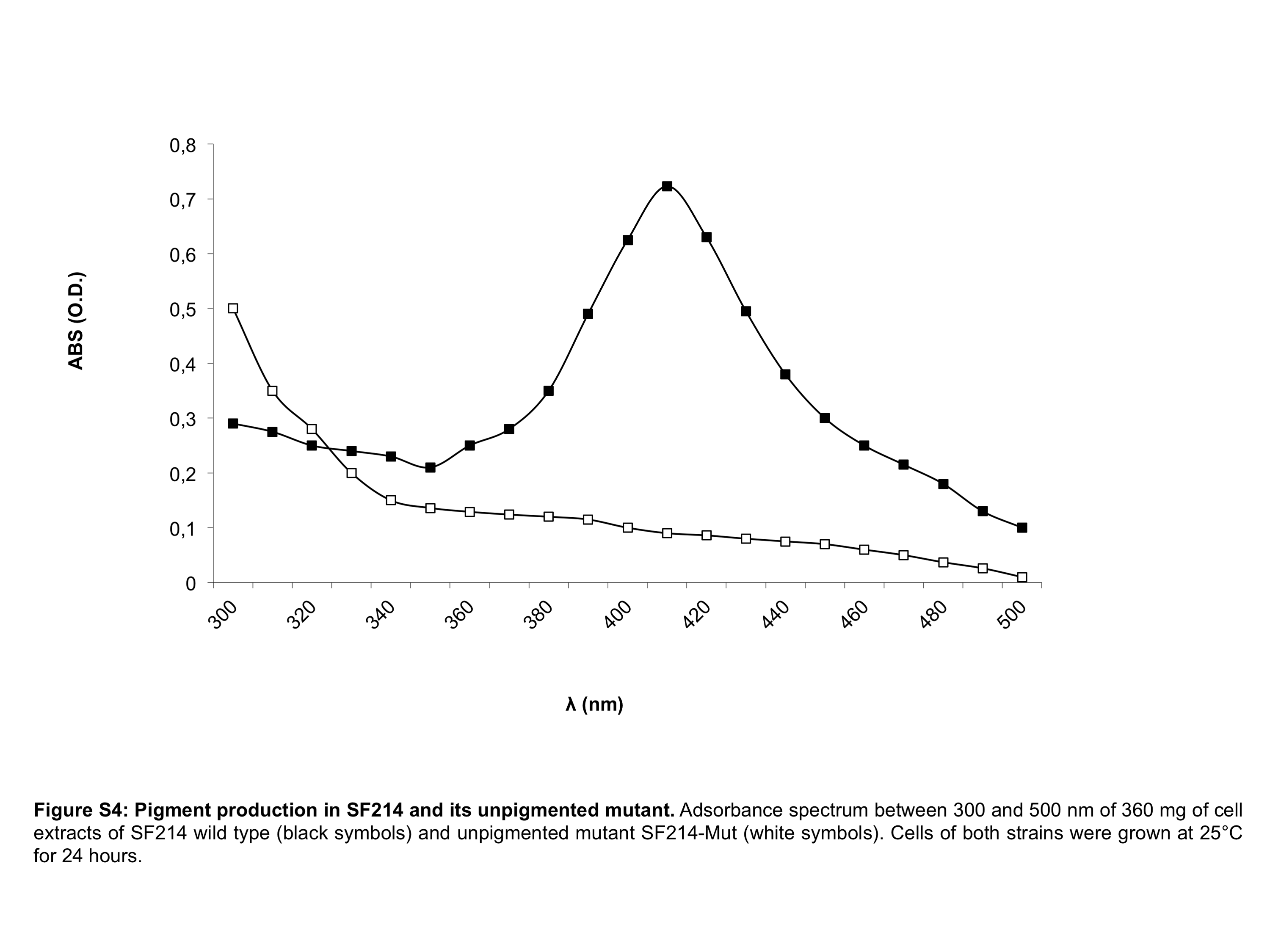

Supplement: Figure S4 — Pigment production in SF214 and its unpigmented mutant. Adsorbance spectrum between 300 and 500 nm of 360 mg of cell extracts of SF214 wild type (black symbols) and unpigmented mutant SF214-Mut (white symbols). Cells of both strains were grown at 25°C for 24 hours. (TIF) [file pone.0062093.s004.tif]
